# Formalized Computer-Aided Handwriting Psychology: Validation and Integration into Psychological Assessment

**DOI:** 10.3390/bs10010027

**Published:** 2020-01-03

**Authors:** Yury Chernov, Claudia Caspers

**Affiliations:** 1 IHS Institute for Handwriting Sciences, 8046 Zurich, Switzerland; 2 Hochschule für Philosophie, 80539 Munich, Germany

**Keywords:** computer-aided handwriting analysis, handwriting psychology, 16PF-R personality assessment, personality traits, validation study, multi-factor psychometric instruments, integration of personality assessment methods

## Abstract

In contrast to traditional researches that involve a manual, non-quantitative, and subjective way of performing handwriting analysis, in the current research, a special computer-aided method of revised handwriting analysis is used. It includes the detection of personality traits via manual quantitative registration of handwriting signs and their automated quantitative evaluation. This method is based on a mathematical–statistical model that integrates multiple international publications on the evaluation of handwriting signs. The first aim is the validation of the revised method against the 16 Personality Factor Questionnaire Revised (16PF-R), which is performed as a self-report personality test by test persons and was developed and researched empirically by Raymond B. Cattell et al. A second aim is the development of an integrated model for assessment including handwriting analysis: when both methods come to the same result on a certain scale, then the construct can be accepted with higher reliability; in contrast, when results are contradictory, they should be regarded as a limitation of each method and raise awareness in the researchers, as these contradictions are a precious source of additional information regarding the complexity, ambiguity, and context specificity of personality traits.

## 1. Introduction

Personality questionnaires based on self-assessment began evolving about a century ago and are still the most common instruments in personality assessment [1,2] for the the time and effort they require and their research economics [3]. In some cases, self-assessment is often the only access to a test person [4]. The 16 PF-R Personality Questionnaire is one of them and is still frequently used today, especially in career counselling and in business for employee testing and selection. Even though questionnaires are nowadays being tested with scientific methods and are improved in terms of performance data and robustness, certain problems of self-assessment questionnaires remain: systematic biases regarding retrospective questions about experiences and frequencies [5], subjectivity of test persons, dependency on context (e.g., purpose of test), tendencies to social desirability (e.g., in the hiring process), and tendencies to the middle or extreme by answers to questionnaire items and ambiguity of understanding questionnaire items.

This is why nonverbal tests are getting more popular. Handwriting psychology, historically known as graphology in its old from, represents a nonverbal testing system for personality traits. It is traditionally used for recruiting personnel, in medicine, forensic, and some other areas. There are certain advantages of this method for psychological assessment:

- Large number of personality traits can be assessed in one procedure.
- Social desirability is totally excluded.
- Writing by hand is a normal and natural activity for most people that excludes the influence of testing situations.
- Handwriting samples can be obtained independently of time, place, and physical presence of the test persons.
- Psychological development of test persons can be assessed when several handwriting samples produced in different ages are available.
- The language of the handwriting sample (valid for European languages) has no influence on the evaluation process.

As with every method, there are also disadvantages, which are mainly valid for the traditional way of handwriting analysis [6,7,8,9,10,11,12]: it can only be performed by specially educated experts and it is a non-standardized, complex, and time-consuming interpretation process with little transparency, recognition, and acceptance of the method in the field of psychology, due to contradictory validation results.

The aim of this study is to use the advantages of handwriting analysis as a nonverbal, additional test system in the framework of personality assessments and to avoid the disadvantages of the traditional method by using the revised, computer-based, and standardized evaluation of handwriting signs with the HSDetect program. By using the HSDetect program, an automatic, computer-based evaluation of 378 personality traits and behavior patterns is done after a manual registration of 544 handwriting signs in a standardized protocol. The content of a handwritten text which is used for the registration of the handwriting signs can be freely chosen by the test persons. In contrast to the traditional method of handwriting analysis, any information in the text or indications that would reveal something about the writer’s personality plays no role in the registration process, since the evaluation of the personal traits with HSDetect is automated and therefore takes place without any interference of an expert. With the revised method, it is not possible for an expert to know any connections between handwriting signs and personality traits included in the program, and therefore the expert cannot, as is common with the traditional method, select suitable personality traits manually from evaluation tables according to her/his experience. Details of the operating principles of the HSDetect program will be presented in Section 2.3 of this article.

In general, the authors are of the opinion that the number of research works with positive validation results is large enough not to disqualify the method and not to reduce their results to “anecdotal evidence” [13] (p. 82). It might be true that there is not enough validation information regarding handwriting analysis, but there is as well not enough scientific evidence and information either to reject it as an assessment method, especially, taking into consideration that the quality of validation studies done so far suffer from “significant methodological negligence” [8] (p. 191) and “many of these studies could be criticized methodologically in terms of measurement of both personality and graphology” [14] (p. 80).

The proposed, revised method with the HSDetect program opens new perspectives due to a formalized approach to handwriting analysis. It implements a clear and literature-based matching of handwriting signs to psychological traits by means of mathematical–statistical modelling and computer-based evaluation. The formalized procedure enables an easy integration of handwriting analysis as an additional useful method into the common personality assessment. It helps to avoid the biased influence of questionnaire tests based on self-evaluation. As a natural step before integration, a validation of the computer-aided method of handwriting analysis has to be done. In the current study, validation against the popular and well-validated test 16 Personality Factor Questionnaire Revised (16PF-R) was selected [15], based on the revised edition of the frequently used 16PF Questionnaire by Raymond B. Cattell, Maurice Tatsuoka, and Herbert Eber.

In summary, the objectives of the present study are as follows:

- Development of a formal validation procedure of computer-aided handwriting analysis.
- Application of this procedure by means of the 16PF-R.
- Development of an integration model for personality assessments including handwriting analysis.

## 2. Materials and Methods

### 2.1. Project Overview

The project covered both the validation study and the model for integrating handwriting analysis into the 16PF-R testing procedure. The last ensures more reliable results for 16PF-R scales. The project consisted of the following steps:

- Step 1: each test person performed the German version of the 16PF-R test and supplied a handwriting sample (free text, one A4-sized page) produced independently in their mother language.
- Step 2: 16PF-R scales were modelled with matching personality traits from the database of HSDetect based on the scale description provided by the developers of the 16PF-R [15]. This model was generated by the authors, and these traits of HSDetect were used for evaluation and validation (cf. steps 5 and 6).
- Step 3: 16PF-R was evaluated by the first author according to the test manual.
- Step 4: handwriting signs of the handwriting samples (originals, no photocopies) were registered manually by the second author (qualified and experienced handwriting expert) with a standardized handwriting protocol consisting of 544 handwriting signs per test person.
- Step 5: handwriting signs obtained with the protocols in step 4 were evaluated algorithmically with the HSDetect program. The result was a list of 378 personality traits and behavior patterns with percentages for each test person.
- Step 6: validation of the 16PF-R test results against modelled scales of step 2 was done.
- Step 7: computer-aided handwriting analysis could easily be integrated into psychological assessment to make the evaluation of psychological constructs more objective.

### 2.2. Participants and Material

The study included 58 participants characterized by three different mother languages and different ages and education levels. Of the participants, 22.4% (13) were male, and 77.6% (45) female; 12 test persons were younger than 30 years of age, 21 were between 30 and 45 years old, 16 between 46 and 60, and 9 over 60 years old.

The test persons were recruited in different areas (work, through colleagues, at events, and via newspaper ads). A required criteria for taking part in the experiment was the ability to write fluently by hand in one’s mother language and the willingness to complete the 16PF-R-questionnaire in German or English. Among the 58 test persons, 44 were German-speaking, 13 Russian-speaking, and 1 English-speaking.

### 2.3. Modelling of Handwriting Evaluation with HSDetect

Traditional validation studies of handwriting analysis have two major problems: the first one is that the procedure of personality trait evaluation used was mostly manual and subjective, although often the experts demonstrated statistically good agreement. The second problem is that the researchers took for practical reasons just small subsets of the handwriting variables and selected a very restricted set of personality traits [14,16,17]. The improved validation procedure proposed in this study and discussed in the current article is based on the computer-aided handwriting analysis called HSDetect and solves both problems. The system is based on the following principles:

- Consolidation and harmonization of different handwriting analysis methods and schools to avoid biased results.
- Formal presentation of all handwriting signs, personality traits, and the relations between them.
- Quantitative registration of handwriting signs and evaluation of personality traits.
- Assurance of evaluation objectivity and reliability.

In HSDetect, the handwriting signs are evaluated manually by experts, and the corresponding psychological traits are calculated algorithmically. Besides the algorithms of the handwriting analysis, HSDetect includes two databases: the database of the handwriting signs and psychological traits with connections between them (the handwriting analysis model) and the database of the evaluation of subjects’ handwriting samples—statistical results that serve as norming data and as a basis for different statistical studies.

Both the handwriting signs and the values of the psychological traits are presented on a continuous scale from 0 to 1. Each psychological trait is mathematically modelled as a function of several handwriting signs. The relations are complex, many-to-many, which means that a handwriting sign relates to several traits, and a typical trait is a function of several signs. A detailed description of the mathematical model of HSDetect can be found in earlier publications [18,19]. The main mathematical principles and an example are explained below:

The trait value of i-th trait t_i_ is modelled by the following function:

(1) t_i_ = y_i_^α^ ∙ r_i_^1−α^

where y_i_ shows the strength of the i-th trait; r_i_ stands for the reliability of the of the evaluation of the trait level; α is a parameter that in the case of this experiment was set to 0.6 on the basis of empirical trials. The strength of a trait y_i_ is the function of the handwriting signs related to this trait:

(2) y_i_ = ∑ a_ij_ ∙ x_j_,

where x_j_ is the value of the handwriting sign registered manually by the expert. The value of x_j_ can range from 0, which means the sign is not present, to 1, which indicates that the sign is always strongly present in the handwriting. The parameter a_ij_ is a weight, which is defined on the basis of the statistical evaluation of multiple sources (publications): the more the sources reporting about an existing relation between the i-th trait and the handwriting j-th sign, the higher the value of a_ij_. The sum is calculated over the set of M_i_ handwriting signs related to the i-th trait.

The reliability r_i_ depends on the number of handwriting signs (n_i_) that are really present (that means x_j_ > 0) in the analyzed handwriting sample. These signs are a subset of M_i_. If we assume that the probability of an error, when we evaluate a trait on the basis of just one handwriting sign, is µ, then the expression for r_i_ is as follows:

(3) r_i_ = 1 − µ^i^,

Assuming µ = 0.25, the value of r_i_ reaches a very high value of 0.9 with eight handwriting signs.

The last step is the normalizing of the trait value t_i_. That is necessary to make different traits comparable to the 16PF-R scales. In a handwriting sample, not every sign is present with the same frequency, which of course depends on language and writing conventions. That could lead to some biases in the trait values, and as a result, some traits would always have higher values than other traits. Normalization avoids this effect. A database of several hundreds of handwriting samples, which were previously evaluated with HSDetect, is used for normalization. Since the distribution of values for most traits is not normal, the following simple formula has to be used instead of the standard z-normalization:

(4) z_i_ = (y_i_ − Y_i_^min^)/(Y_i_^max^ − Y_i_^min^),

where z_i_ is the normalized value of the i-th trait; Y_i_^min^ and Y_i_^max^ are the correspondingly minimal and maximal absolute values of the i-th trait (derived from the databank).

Below, the theory of the above-described mathematical–statistical basis of the HSDetect program is illustrated by an example. In Table 1, the mapping of the trait “responsiveness” (empathy, consideration) with the connected handwriting signs as reflected in expression (2) is presented.

**Figure 1:**
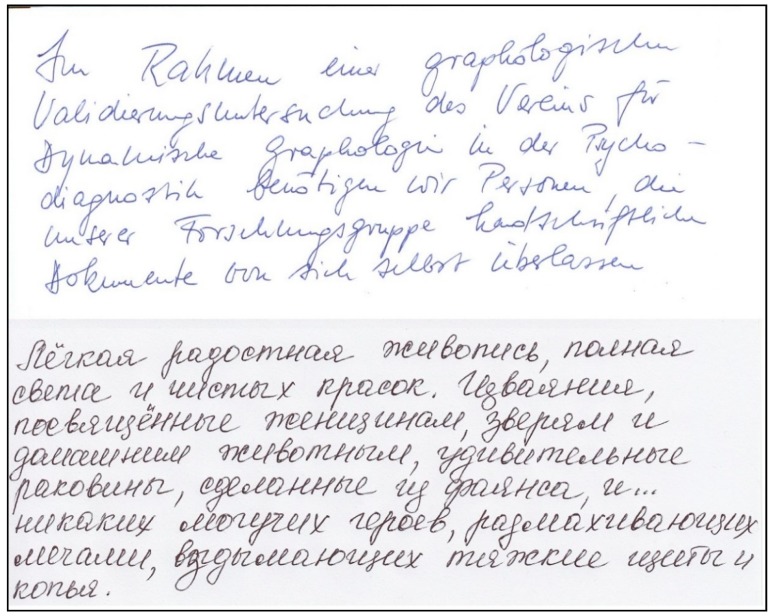
Handwriting samples with high responsiveness.

Two examples of handwriting samples (cuttings) with high responsiveness are shown in Figure 1.

It can be clearly seen how different handwriting signs affect the trait level in the model. In practice, it is very rare that all of the possible signs per trait are present in a handwriting sample. Vice versa, often “contradictory” signs, those that point to an opposite, negative pole of the trait, are present as well. In Table 2, the model of the negative pole (“unresponsiveness”) is shown.

**Figure 2:**
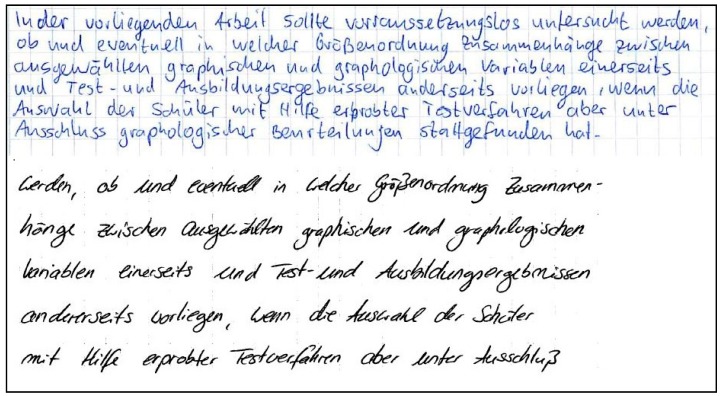
Handwriting samples with low responsiveness.

Two examples of handwriting samples (cuttings) with low responsiveness from our experiment are shown in Figure 2.

**Figure 3:**
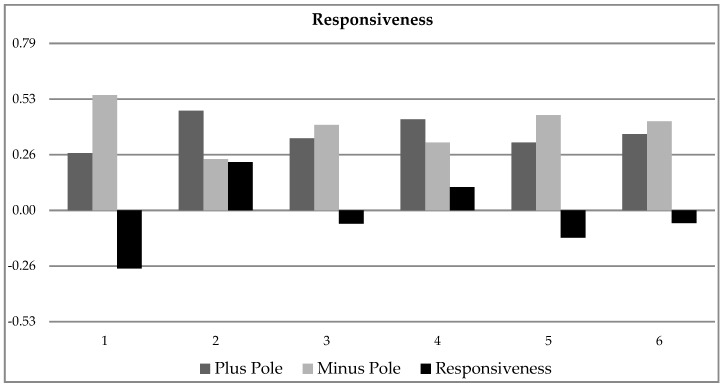
Example of trait Responsiveness for the first six subjects.

Since, in one sample, it is very likely to find signs related to both sides of a pole of a trait, it is always necessary to look at the difference between the plus and minus poles, which is calculated in expression (5). Figure 3 presents the result of the evaluation of both poles of the trait “responsiveness” and their difference. This an example of 6 out of 58 test persons. All test persons were evaluated with expression (4).

### 2.4. Scale Modelling of 16PF-R with Handwriting Analysis

In many validation studies with handwriting analysis and psychometric tests, modelling of test scales or factors is insufficiently implemented in the sense of oversimplification [9,20]. In the current project, each scale of the test was modelled by a thoroughly selected set of psychological traits based on the description of the 16PF-R test’s authors [21], which is presented in Table 3. The values of these traits were evaluated by handwriting analysis with HSDetect. Therefore, the path looked as follows: 16PF-R test scales -> corresponding psychological traits from HSDetect. In many former corresponding validation studies, the authors would directly map handwriting signs onto questionnaire test scales [16,17,22]. The number of manually involved signs is as well strongly restricted. The indirect integration of handwriting signs by modelling of the questionnaire test scale though traits of the HSDetect program is the uniqueness of this experiment and seems to be a smarter approach to effective validation results. This approach is practically possible only with a computer-aided procedure.

As already mentioned, in handwriting analysis, the complex relations between signs and traits could lead to unspecific results. Therefore, it is important to include into the modelling process not only the positive pole of a trait of a questionnaire test scale but also the negative one. For instance, for scale A (“warmth”), both already mentioned poles, “responsiveness” and “unresponsiveness”, are necessary, or for scale C (“emotional stability”), in addition to “emotional stability”, also the negative pole, “emotional instability”, must be taken into consideration.

Therefore, the formal presentation of a modelled scale value is as follows:

(5) S = ∑z_i_^+^/n + −∑z_j_/n^−^,

where S is the scale value; z_i_^+^ is the value of the i-th trait of the positive pole of the scale; z_i_^−^ is the value of the corresponding i-th trait of the negative pole; n^+^ and n^−^ are the numbers of traits of the positive and negative poles. The positive pole represents the high scores of a test scale [15,21], and correspondingly, the negative pole represents the low scores of a test scale.

Each scale of the 16PF-R is modelled with expression (5). The traits presented in Table 3 were calculated with expressions (1)–(4) for the evaluation of each test scale. The quality of the modelling of the scales was verified with Cronbach’s Alpha [23]. To estimate the Cronbach’s Alpha value. we used the norming data of HSDetect with several hundreds of handwriting samples and their evaluations.

### 2.5. Method of Validation

For validation of the computer-aided handwriting analysis, the calculated 16PF-R scales were compared with the results of the questionnaire test to verify the agreement between them. It is very important how a comparison procedure is designed. Often, researchers only use standard correlation as a simple and easy applicable method [24]. According to the authors’ opinion, this method is inappropriate for multi-factor psychometric instruments like handwriting analysis.

First, both methods are not really comparable, as handwriting analysis is a foreign-assessment method, and 16PF-R is a self-assessment method. It is well known that a gap between self- and foreign-assessment exists, which should not be underestimated [25,26,27].

Secondly, although the data of both 16PF-R and handwriting analysis are quantitatively generated, a reasonable interpretation cannot be so mathematically exact for individual test persons (as occurred in our experiment). Only statistically calculated variables (like mean or deviation) of large sample sizes can be purely mathematically compared.

Thirdly, the nature and number of items in 16PF-R and handwriting analysis are so different that the two types of results can only be formally compared with a system that reduces the complexity of these differences. Besides, stochastic influences of different factors on both systems are high.

To allow a reliable agreement check, the results of the 16PF-R-factor presentation of both the test and the handwriting analysis were mapped on a scale with three intervals: positive (strong domination of the positive scale pole), negative (strong domination of the negative scale pole), and neutral (absence of strong domination of either pole). The zone boundaries were individually defined for each scale according to statistical data.

In the 16PF-R questionnaire, each question is mapped on one scale, and each answer (with a choice of three variants) has its weight (for 16PF-R, the weights are 1, 2, or 3). That is typical for a questionnaire test. Thus, each test scale has its minimal and maximal scores depending on the number of related questions. For instance, scale A ranges from 9 to 27, since there are 9 questions related to it. For the zone-based comparison, the negative zone ranges from 9 to 15, the neutral zone from 15 to 21, and the positive zone starts at 21 and finishes at 27. Also, for scale G, the negative zone is from 11 to 18, the neutral zone from 18 to 26, and the positive zone from 26 to 33. This assumes that the distribution is close to uniform, which is often the case.

In the same way, zones for the handwriting model are defined. However, the boundaries are not predefined but depend on the results of the evaluation of all subjects. Predefined zone boundaries could result in strong biases: their theoretical values can change from −1.0 to +1.0. Practically, the distribution for each trait and, correspondingly, every scale might vary very strongly and it is far from being uniform.

### 2.6. Method of Integration

Traditionally, psychological assessments prefer the use of direct self-reports in form of standardized questionnaires, whereas some researchers prefer the use of indirect, nonverbal testing methods, as they are interested in test results without the effects of language interpretation problems, cultural differences, and faking while filling out questionnaires [25,28].

From the perspective of the test persons, it is often not easy to fill out questionnaires, as they start reflecting about “consistency–inconsistency” or the “organizations’ expectations” during the answering process [25] (p. 448). Other mentioned obstacles with answering questionnaire tests are the use of extreme versus middle responses, the “lack of ‘ambiguous’ as a response option”, and the lack of “information about the context” for some items [25] (p. 446).

The mentioned disadvantages of self-assessment questionnaires can be compensated by the integration of handwriting analysis in the assessment. The integration enables the improvement of evaluation objectivity of the psychological constructs, which are presented in the current research by 16PF-R scales: when both 16PF-R and handwriting analysis come to the same result on a certain scale despite different response styles of the test persons, then this construct can be accepted with higher reliability. When results are contradictory, then they should be regarded as a limitation of each method and raise awareness in the researchers, as these contradictions are a precious source of additional information regarding the complexity, ambiguity, and context specificity of personality traits.

## 3. Results

The summary of the evaluation of 58 test persons for each scale is shown in Table 4. It shows the numbers of agreements, neutral results, and contradictions (Contr.). Agreement per test person means that the results are in the same zone, whereas contradiction means that they are in opposite zones. Neutral represents the combinations with adjoining zones.

To check statistical significance, binomial distribution with three outcomes can be used. For 58 test persons and the standard significance level of 0.05, the critical number of outcomes is 25. From Table 4 we can conclude that 14 of 16 scales showed a good statistical agreement. No scales showed statistically significant disagreement.

## 4. Discussion and Conclusions

In general, it can be concluded that the zone-based comparison instead of a simple correlation showed reasonably positive results between 16PF-R and handwriting analysis. The assumption that people who can be labelled as “perfectionists” (Q3) on one method should also appear “perfectionists” on all other methods is grounded in a one-dimensional view of the human nature that holds it is impossible for people to be a “perfectionist” and a “non-perfectionist” at the same time. A variety of different contexts can be identified that illustrate that personality traits are not uniform and one-dimensional across personality. In addition, method variance [29] due to distinctions between the methods discussed in Section 2.6 has to be considered as a factor not to be neglected. Therefore, the strengths and limitations of each assessment method (questionnaire self-assessment test and foreign-assessment handwriting analysis) should be recognized and appreciated. The different response styles of the 16PF-R test and the handwriting analysis in combination can provide useful information about personality traits, as both methods together can consistently illuminate the full scope of any personality construct.

On the basis of our findings, future research could confront the test persons with the self-concepts determined by the results of the 16PF-R and the handwriting analysis and find out which concepts in which specific contexts fit best to the self-perception of the test persons. With these results, the 16PF-R scales modelled with traits from handwriting analysis can be differentiated context-specifically. This will contribute to the differentiation of the 16PF-R scales and to the improvement of scale modelling (cf. Table 4), while taking into account the dynamic and stable aspects of self-perception.

The presented unique approach of revised, computer-assisted handwriting analysis can also be implemented in big-data research, as new connections between handwriting signs and personality traits could be determined in future studies, considering the more than 500 recordable handwriting signs in the HSDetect database and results from various verbal and nonverbal personality tests.

## Figures and Tables

**Table 1:** Handwriting signs for trait “responsiveness”.

No	Handwriting Sign	Parameter a_ij_
1	Connection form—garlands or threads	0.193
2	Normal or strong right slant	0.130
3	High fullness of letters (especially middle zone)	0.105
4	Pasty stroke formation	0.084
5	Last letters have lighter pressure	0.072
6	Letter size in words is tapering	0.060
7	Diacritic marks are bound to the letter and are irregular	0.043
8	Lead-out stroke of last letters is long ascending or horizontal	0.038
9	Larger middle zone	0.038
10	Connected handwriting	0.038
11	Legible signature	0.035
12	Irregular handwriting	0.029
13	Ovals open at top	0.027
14	Wide letters	0.018
15	Legible handwriting	0.018
16	Small letter size	0.018
17	Diacritic marks—stronger pressure, emphasized, heavy	0.013
18	Average word spacing	0.009
19	Return stroke of lower zone is rising to the right of stem	0.009
20	Left margin is wide	0.009
21	Narrow line spacing	0.009
22	Rising line direction	0.009

**Table 2:** Handwriting signs for trait “unresponsiveness”.

No	Handwriting Sign	Parameter a_ij_
1	Thin, sharp stroke formation	0.145
2	Angular connection form	0.145
3	Emphasized last letters	0.082
4	Elongated or threading letter form	0.095
5	Uneven, not controlled margins	0.065
6	Narrow letters (small width)	0.063
7	Smaller middle zone	0.063
8	Horizontal line direction	0.063
9	Slant in words becomes smaller	0.060
10	Narrow margins	0.050
11	Thin handwriting form	0.038
12	Vertical or left slant	0.057
13	Small letter size	0.038
14	Narrow word spacing	0.019
15	Slow speed	0.019

**Table 3:** Scale modelling of the 16 Personality Factor Questionnaire Revised (16PF-R) with handwriting analysis.

Scales	Scale Label	Positive Pole	Negative Pole
A	Warmth	responsiveness, compulsiveness, need of contacts, lack of reserve	unresponsiveness, reserve, keep distance from the outer world
B	Reasoning	shrewdness, logical thinking, abstract thinking	inertia of thinking, absence of logic, concrete thinking
C	Emotional Stability	emotional stability, stability under stress, poise	emotional instability, cannot keep stability under stress, irritability
E	Dominance	dominance, persuasiveness, conflicted character, self-assuredness, obstinacy	softness, diplomacy, inability to persuade, self-uncertainty
F	Liveliness	lack of reserve, infantilism, naturalness, vanity, lack of restrains, loquaciousness	moderation, maturity, reticence, artificiality, reserve, inconspicuousness
G	Rule-Consciousness	conventionality, resignation, politeness, responsibility, resistance	eccentricity, irresponsibility, independence, impoliteness, lack of resistance
H	Social Boldness	extroversion, vanity, interpersonal skills, impudence	unsociability, inconspicuousness, diffidence, withdrawnness
I	Sensitivity	emotionality, sensibility, sentimentality, receptiveness	good judgement, cynicism, insensitivity, insusceptibility
L	Vigilance	skepticism, carefulness, mistrust	naivete, imprudence, enthusiasm
M	Abstractedness	creativity, idealism, imaginativeness, poor judgement, inattentiveness	good judgement, lack of imagination, reasoning, lack of creative ability, attentiveness
N	Privateness	insincerity, tactfulness, nondisclosure	sincerity, tactlessness, indiscretion
O	Apprehension	conscience, melancholia, self-uncertainty, anxiety, inner conflict	integrity, unconcern, unscrupulousness, self-assuredness, zest for life
Q1	Openness to Change	curiosity, dynamics, versatility, fussiness, solidity, lack of resistance	resistance, conservatism, lack of curiosity, narrow field of interests
Q2	Self-Reliance	independence, individualism, self-sufficiency, self-dependence	dependency, cooperativeness, need of contacts
Q3	Perfectionism	neatness, meticulousness, methodicalness, zealousness	negligence, disorderliness, inaccurateness, scrappiness
Q4	Tension	irritability, impatience, nervousness, self-control giving way to excitement	patience, quietness, poise, control of initial impulses

**Table 4:** Result summary of zone agreement of 16PF-R and HSDetect (n = 58).

Result	A	B	C	E	F	G	H	I	L	M	N	O	Q1	Q2	Q3	Q4
Agreement	25	30	26	26	28	31	32	26	17	29	26	31	30	26	19	32
Neutral	18	15	18	19	17	19	10	20	24	17	23	16	18	20	24	15
Contr.	15	13	14	13	13	8	16	12	17	12	9	11	10	12	15	11
∑	58	58	58	58	58	58	58	58	58	58	58	58	58	58	58	58
